# Contribution of household dishwashing to microplastic pollution

**DOI:** 10.1007/s11356-023-25433-7

**Published:** 2023-01-26

**Authors:** Daniel Sol, Andrea Menéndez-Manjón, Sofía Carrasco, Jacinto Crisóstomo-Miranda, Amanda Laca, Adriana Laca, Mario Díaz

**Affiliations:** Department of Chemical and Environmental Engineering, University of Oviedo, C/ Julián Clavería s/n, 33006 Oviedo, Spain

**Keywords:** Microplastics, Tap water, Dishwasher, Plastic lunch box, Detergent, Pollution

## Abstract

At household level, clothes washing has been recognised as an emitter of microplastics (MPs) into the environment and it is supposed that dishwashing is also a source of MPs, although little attention has been paid so far. In this work, the emission of MPs released from dishwashing procedures at household level has been studied. The effect of different parameters such as time, temperature and type of detergent has been analysed. In addition, the MP content of tap water has been evaluated in order to determine its contribution to the MPs in dishwasher effluent. Results showed that when the dishwasher was operated empty with a pre-wash programme (15 min and room water temperature), between 207 and 427 MPs were released per load (3 L), whereas this value increased notably with an intensive programme (164 min and water at 70 °C) (1025–1370 MPs per load, 15 L), which highlighted the effect of temperature and time on MP release. Additionally, when a polypropylene lunch box was washed, the number of MPs released increased by 14 ± 3 MPs and 166 ± 12 MPs of total. Finally, the influence of the use of detergent with the dishwasher empty and containing lunch boxes has been studied. With detergent, 35–54% more MPs were released from dishwasher accessories, whereas no additional release took place from lunch boxes. This work shows for the first time the important contribution of domestic dishwashing to MP pollution and the environmental benefits of using more environmentally friendly materials in both dishwashing machine accessories and food utensils.

**Supplementary Information:**

The online version contains supplementary material available at 10.1007/s11356-023-25433-7.

## Introduction

Microplastics (MPs) can be released into the environment as ‘primary microplastics’ (from cosmetics, personal care products, paints, washing textiles, etc.) or ‘secondary microplastics’ (originating from industries, agricultural activities, fishing, tyre wear and also at household level) (Auta et al. 2017; Boucher and Friot 2017; Li et al. 2016). Most of these microparticles end up in sewage systems which are one of the main culprits of MP release into the environment (Horton et al. 2017; Padervand et al. 2020; Petersen and Hubbart 2021; Xu et al. 2020). Certainly, wastewater treatment plants (WWTPs) can remove more than 90% of MPs from wastewater, but this is insufficient, since millions of microplastics are still discharged into the environment every day by each WWTP (Ali et al. 2021a, b; Masiá et al. 2020; Sol et al. 2020; Menéndez-Manjón et al. 2022). More specifically, it has been estimated that a WWTP releases between 0.01 and 2.97 × 10^2^ particles per liter of effluent (Ali et al. 2021a, b; Liu et al. 2021).

MPs have been detected at household level in items of clothing and furnishing (Suaria et al. 2020). Washing textiles has, in fact, been described as the most important source of primary microplastics, especially microfibres, found in wastewaters (Boucher and Friot 2017; Sol et al. 2022). Browne et al. (2011) reported that an item of clothing can release more than 1900 fibres per wash, while another study showed that one polyester fleece garment can emit around 1.1 × 10^5^ fibres in only one wash (Almroth et al. 2018) and 2.1 × 10^5^ and 1.3 × 10^7^ microfibres can be released per kilogram of polyester and cotton textiles, respectively (De Falco et al. 2019; Sillanpää and Sainio 2017). Furthermore, it has been reported that the use of detergents can increase the emission of microplastics from clothing by up to 1.8 × 10^7^ fibres (De Falco et al. 2018a, b). Periyasamy (2021) studied the effect of different factors in jeans washing and found that the higher the temperature and the higher the spin speed, the higher the microfibre emission. Additionally, household activities emit MPs to the atmosphere and concentrations of 4–59 fibres/m^3^ have been detected in indoor environments, whereas this value was notably lower outdoors (0.3–1.5 fibres/m^3^) (Dris et al. 2017). All these data clearly indicate that household activities can be an important source of MPs.

Another important aspect at household level is the use of tap water for personal domestic use. In particular, when the presence of MPs was analysed in effluents from drinking water treatment plants (DWTPs), concentrations varied from *not detected* to 6614 MPs/L (Sol et al. 2021). This reflects a high variability in the number of MPs that can be found in tap water. For example, China and the Czech Republic were shown to have very high MP concentrations (1296–6614 MPs/L) (Pivokonský et al. 2018, 2020; Shen et al. 2021; Wang et al. 2020), whereas much lower MP concentrations were detected in studies carried out in Germany, Spain and Thailand (Chanpiwat and Damrongsiri 2021; Dalmau-Soler et al. 2021; Mintenig et al. 2019).

The dishwasher has become a common household appliance. In fact, 80% of households in the USA and 44% in Europe have one. Regardless of the brand, a dishwasher consists of 40–50 parts made of different materials. Stainless steel accounts for more than half of the mass by weight (53%) of the dishwasher, followed by the mastic sound-dampening insulation that surrounds the tub (15%), while plastic parts account for 24%. The remaining 8% corresponds to printed circuit board, pulp, wiring harness, among other things (Porras et al. 2020; Venkatesh 2022). The present study is aimed at contributing to widen the knowledge on household activities as source of MPs by analysing dishwashing as another possible source. As far as we know, no studies investigating the release of MPs from dishwashers have been carried out despite the fact that the dishwashing machine accessories and some of the utensils washed are often made of plastic. The effect of different parameters has been evaluated, i.e., time, temperature, use of detergent and the washing of plastic lunch boxes, with the aim of determining which factors are the most relevant, as well as the importance of these common household appliances in the release of microplastics into the aquatic environment.

## Materials and methods

All the distilled water used for washing the materials and experimentation was previously filtered through cellulose acetate filter (Ahlstrom-Munksjö, 0.45 μm) to avoid contamination of samples with MPs.

### Microplastics in tap water

Tap water samples (10 L) taken at the Faculty of Chemistry in Oviedo (Spain) were filtered through four overlapping sieves with a mesh size of 500, 250, 100 and 20 μm (CISA Sieving Technologies). The solids retained on each of the sieves were washed away with filtered distilled water. The water recovered was filtered under vacuum (0.7-μm glass microfibre, Whatman), so MPs were retained in the filters. Tap water samples were collected at 9 different dates in February, March and November 2021, coinciding with the dates of experimentation (see Table 1).

**Table 1:** Summary of the dates and number of samples taken for each set of experiments. Samples F22, F24 and F26 were collected in February 2021, whereas samples M23, M24 and M25 were collected in March 2021. Finally, samples N2, N3 and N8 were collected in November 2021. Sample collected ( +) and not collected (-)

	Samples									
	F22	F24	F26	M23	M24	M25	N2	N3	N8	Number of samples
Tap water	+	+	+	+	+	+	+	+	+	9
Pre-wash	+	+	+	+	+	+	-	-	-	6
Intensive	+	+	+	+	+	+	+	+	+	9
Pre-wash + plastic lunch boxes	-	-	-	+	+	+	-	-	-	3
Intensive + plastic lunch boxes	-	-	-	+	+	-	+	+	+	5
Detergent	-	-	-	-	-	-	+	+	+	3
Intensive + detergent	-	-	-	-	-	-	+	+	+	3
Intensive + plastic lunch boxes + detergent	-	-	-	-	-	-	+	+	+	3

### Analysis of microplastic content in detergents

The content of MPs in three commercial brands of dishwasher detergents were analysed. Two of the detergents are supplied as cubes, whereas the third is sold as pods (Figure S1). The chemical compositions of the detergents are detailed in Table 2.

**Table 2:** Chemical composition of the three commercial brands of dishwasher detergents used according to the manufacturers’ information

	Chemical composition
Detergent 1 (Finish)	5– < 15% oxygen-based bleaching agents, < 5% phosphonates, non-ionic surfactants, polycarboxylates. Contains enzymes (subtilisin, amylase). Contains perfumes
Detergent 2 (Presto)	5–30% oxygen-based bleaching agents, < 5% non-ionic surfactants, polycarboxylates. Contains enzymes and perfumes
Detergent 3 (Fairy)	Oxygen-based bleaching agents, < 5% phosphonates, 5–15% non-ionic surfactants, polycarboxylates, enzymes, perfumes, citronellol, limonene, linalool

To recover the plastic particles, each cube/pod was treated with 30 mL of hydrogen peroxide (50%, VWR) for 24 h to oxidize the organic compounds present in the samples. Then, samples were placed in an oven at 90 °C until they were dry (approximately 5 h). Once dried, 80 mL of a prepared solution of zinc chloride with density 1.6 g/mL (97% purity, VWR) was added to the samples in a 120-mL beaker. MPs were isolated from the supernatant by means of filtration under vacuum using a glass microfibre filter (Whatman, diameter 47 mm, pore size of 0.7 μm). To recover all the MPs, this step was repeated twice, adding zinc chloride solution to the pellet. MPs retained in the filter were finally washed out using filtered distilled water. The detergent with the highest MP content was selected to carry out the dishwashing assays.

### Dishwashing processes

Washing tests were performed in a medium load dishwasher (Beko DFS28021W) made of stainless steel and polypropylene reinforced with 20% talcum (PP 20 T). All the plastic accessories are grey, except for the upper and lower spray arm which are purple (Figure S2).

Two washing programmes were tested: the pre-wash programme (15 min at room water temperature with an average water consumption of 3.4 L) and the intensive programme (164 min at 70 °C with an average water consumption of 15.2 L). Different tests with and without detergent and with the dishwasher full and empty were carried out. An overview of the experiments conducted in this work is shown in Table 1.

During the experiments, the water that drained from the dishwasher was collected in plastic containers, measured with a graduated cylinder and then filtered through four overlapping sieves with a mesh size of 500, 250, 100 and 20 μm (CISA Sieving Technologies). The solids retained on each of the sieves were washed away with filtered distilled water, and, after that, the water was filtered under vacuum (0.7-μm glass microfibre, Whatman), so MPs were retained in the filters.

The plastic containers used were made of plastic, so a control experiment has been carried out in triplicate in order to be sure that the number of MPs released from them was despicable. Five liters of filtered distilled water was added to each container and shaken vigorously for 1 min. After this time, the water was filtered and analysed by stereomicroscopy. The number of MPs emitted from each container was 11.3 ± 2.1 MPs/container, which means an increment lower than 1% and 5% in the number of MPs in intensive and pre-wash, respectively.

Prior to the experiments here shown, the brand-new dishwasher was conditioned by means of two pre-washes and between 4000 and 5000 MPs were obtained in these trials. This high number of MPs emitted during these first washings was due to the manufactured plastic parts of the dishwasher, which may present particles adhered to the surface that can be washed away during the dishwashing procedure.

### Plastic lunch boxes

Six equal square plastic lunch boxes, with a capacity of 0.75 L and dimensions of 12 × 12 × 8 cm, made of polypropylene (the containers were transparent, whereas the lids were blue) were employed in the tests carried out with the dishwasher full (Figure S3).

### Microplastic analysis

MPs retained on the filters obtained from the different assays were examined under a semiautomatic stereomicroscope (Leica M205FA) equipped with a high-resolution colour digital camera (Leica DFC310FX) with a maximum resolution of 1392 × 1040 pixels (1.4-Mpixel CCD). The quantification and analysis of colour and shape of MPs were carried out using this equipment.

The type of each polymer (chemical composition) was determined by Fourier transform infrared (FTIR) spectroscopy (Varian 620-IR y Varian 670-IR). After a first visual count, 30% of the particles present in the filter were analysed by FTIR, resulting in approximately 70% microplastics. Subsequently, an extrapolation of the data was made according to the classification by colour and shape collected in the stereomicroscope. The mid-infrared (4000–400 cm^−1^) was used to analyse the samples, this being the most typical range at which bands of plastic are identified. The list of absorption bands of polymers described by Jung et al. (2018) was used to identify functional groups and molecular composition of polymeric surfaces.

In the present study, the quality assurance and quality control (QA/QC) from sampling to the MP quantification has been carried out following the guidance by Brander et al. (2020).

## Results and discussion

### MP content in tap water

**Fig. 1:**
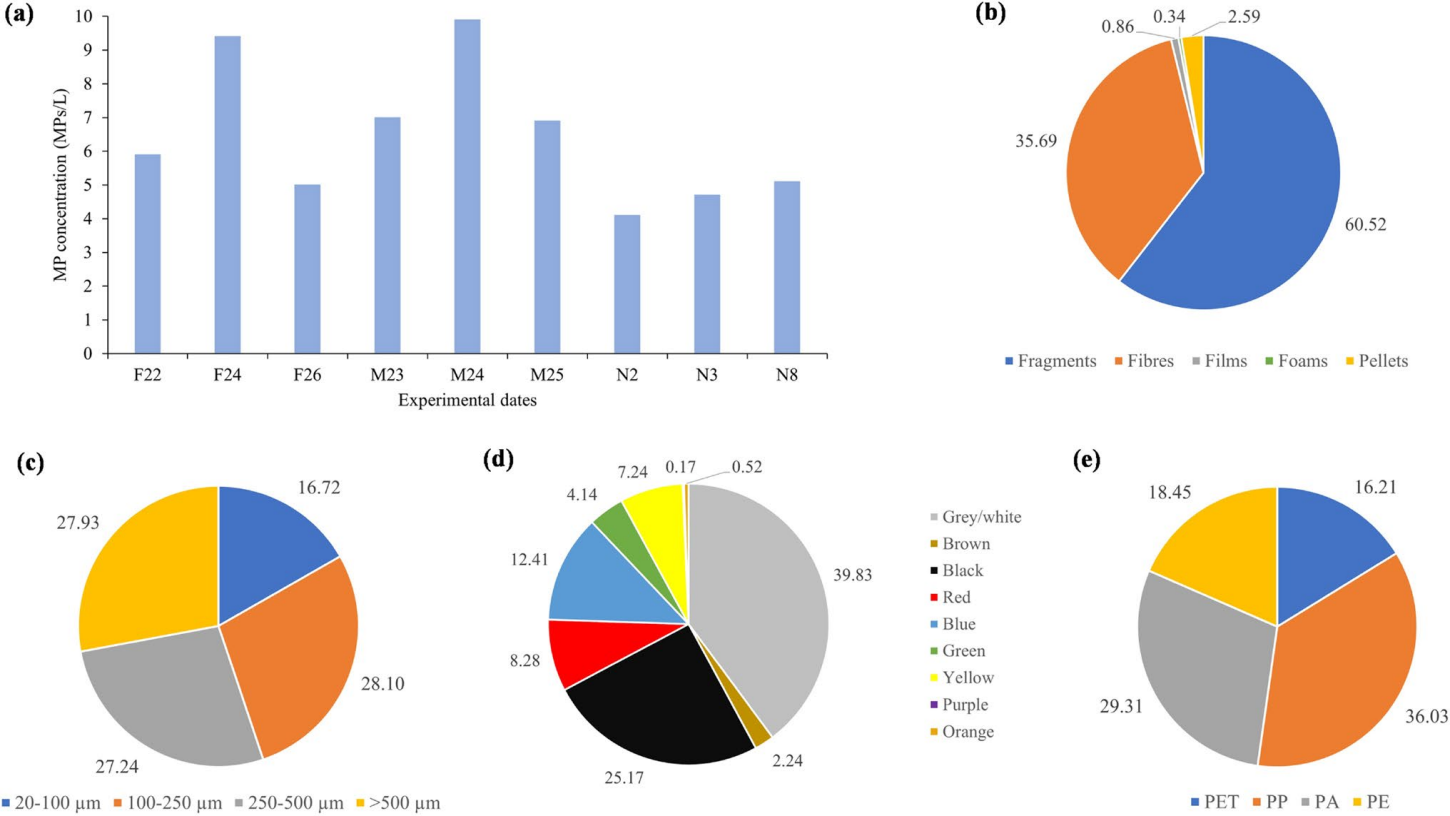
Main characteristics of MP contained in tap water from Oviedo (Spain). **a** MP concentrations found in different samples, **b** average MP shape distribution, **c** average MP size distribution, **d** average MP colour distribution and **e** average MP chemical composition distribution. Samples were taken in 2021 (F22, F24 and F26 in February; M23, M24 and M25 in March; N2, N3 and N8 in November)

Tap water samples were taken at several dates from the same water supply point to which the dishwasher was connected and MP concentrations were analysed as shown Fig. 1a. The concentration of microplastics varied between 4.1 and 9.9 MPs/L, with an average value of 6.4 ± 2.1 MPs/L (20–5000 μm), showing that no notable seasonal variations occurred. These concentrations are in accordance with previous studies carried out by Kosuth et al. (2018) and Almaiman et al. (2021) that found an average value of 5.5 MPs/L (≥ 2.5 μm) when analysing tap water from 14 countries worldwide and 4.7 MPs/L (25–500 μm) from Saudi Arabia, respectively. However, other authors have observed higher MP concentrations, e.g., 194–438 MPs/L in Brazil (6–50 μm) (Pratesi et al. 2021); 27–97 MPs/L (19–4200 μm) in Finland, France, Germany, Japan and USA (Mukotaka et al. 2021) and 44–344 MPs/L (1–5000 μm) in China (Shen et al. 2021; Tong et al. 2020). There are also other studies that found lower values than those obtained in the present work, ranging between not detected and 1.6 MPs/L (≥ 10 μm) (Mintenig et al. 2019; Strand et al. 2018; Uhl and Svendsen 2018; Weber et al. 2021; Zhang et al. 2020). As commented, the range of MP concentrations found in tap water around the world is wide and concentrations here found can be considered fairly low within the usual ranges. In addition, the sizes analysed in the literature were very variable, causing in many cases the underestimation of MPs.

With respect to the shape, fragments were the most common type of MP found in the analysed tap water (60.5%), followed by fibres (35.7%) and pellets at a rather lower percentage (2.6%) (Fig. 1b). The same trend has been observed by other authors, who only found fragments, fibres and pellets, the first being the most frequent form of MP (Mukotaka et al. 2021; Pivokonský et al. 2018; Tong et al. 2020).

Regarding particle size, in all cases, there was a fairly homogeneous distribution and an average of 83% of the total corresponded to MPs bigger than 100 μm (Fig. 1c). On the contrary, Mintenig et al. (2019) found that in tap water from Germany, all MPs were in the range of 50–150 μm and Pivokonský et al. (2018) did not find MPs bigger than 100 μm in tap water samples from the Czech Republic. Tong et al. (2020) detected MPs bigger than 300 μm in tap water samples from China, in agreement with results obtained in the present work, in which around 55% of the MPs were identified as being larger than 250 μm.

Most MPs found here were white/grey and black (65%), followed by blue (12%), red (8%) and yellow (7%) (Fig. 1d). These colours are consistent with those observed by Zhang et al. (2020) from samples of China. With respect to the composition, only four polymers have been identified in the tap water samples, these being polypropylene (PP), the most common (36%), followed by polyamide (PA, 29%), polyethylene (PE, 18%) and polyethylene terephthalate (PET, 16%) (Fig. 1e), which are also the polymers mainly detected in tap water by other authors (Shen et al. 2021; Tong et al. 2020).

### Emission of MPs from a dishwashing machine

As commented above, two washing programmes with different lengths and water temperatures were selected for the experiments with the dishwashing machine: pre-wash (15 min at room water temperature) and intensive (164 min at 70 °C). To determine the number of MPs that are released from the dishwasher, washing experiments with the empty dishwasher machine have been carried out.

**Fig. 2:**
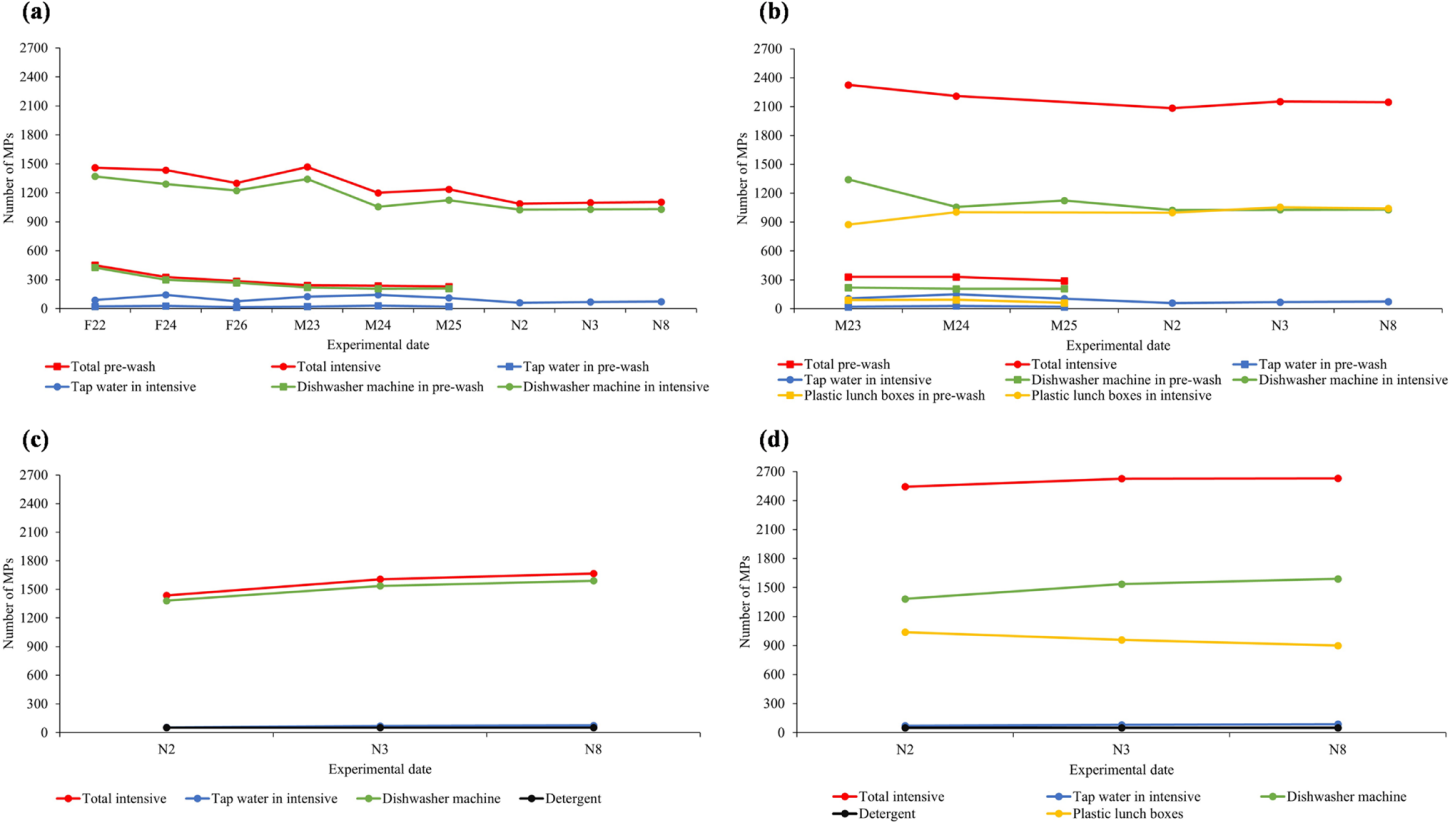
Number of MPs emitted during the different experiments carried out. **a** Empty dishwashing machine, **b** with plastic lunch boxes, **c** empty dishwashing machine with detergent and **d** with detergent and plastic lunch boxes. Samples were taken in 2021 (F22, F24 and F26 in February; M23, M24 and M25 in March; N2, N3 and N8 in November)

Figure 2a shows the total number of MPs found in the effluent from the pre-wash and intensive washing programmes. As expected, because of the different time and temperature, the total number of MPs emitted during the intensive washing is higher than in pre-washing, ranging between 1087 and 1468 MPs (1265 ± 158 MPs) and 230 and 450 MPs (295 ± 84 MPs), respectively. It should be noted that within this total number, there are MPs that originate from the tap water. The contribution of MPs from tap water depends on the volume of water employed during the washing process, so the higher the volume, the higher the number of MPs emitted. Tap water contributions have been estimated by considering the MP concentration measured in the tap water at each experimentation date and the amount of water drained during each washing. Then, the number of MPs released from the dishwashing machine was calculated, these being in all cases more than 90% of the total MPs measured in the effluent. This means that, in all cases, the number of MPs released from the machine accessories was at least 10 times greater than the MPs contained in the tap water.

Comparing results obtained on different dates (Fig. 2a), it can be observed that for the pre-washing, the number of MPs released from the machine in February experiments was higher than that for the experiments carried out in March. The same was observed for the intensive washing cycle, with more MPs released in February experiments and M23 and with almost constant results for the rest of the experiments. It seems that the new dishwashing machine released more MPs during the first washings and after a certain number of washing cycles, the numbers of MPs released during each programme was almost the same. This is in line with the comments in the ‘Dishwashing processes’ section, describing how the dishwasher was conditioned and emitted 4000–5000 MPs by entrainment of particles adhered to the surface of manufactured plastic parts of the dishwasher, considering that water is the main transport vector for MPs (Ali et al. 2021a, b; Uzun et al. 2022; Vivekanand et al. 2021).

In terms of MP concentrations, it can be observed that in the pre-wash, this value decreased from 118 MPs/L to a stable value ranging from 75 to 71 MPs/L. After a series of washes, it can be seen that during the pre-wash cycle, the dishwasher emitted a concentration of 66–64 MPs/L, while tap water contributes 5–10 MPs/L (Figure S4a).

As can be observed in Fig. 2a, the number of MPs emitted from the machine accessories during the intensive programme was notably higher than that obtained in pre-washing, 1025–1370 MPs (1166 ± 143 MPs) and 207–427 MPs (272 ± 85 MPs), respectively. This clearly indicates that longer times and higher temperatures cause more degradation of the plastic parts inside the dishwasher (basket, detergent and rinse aid dispenser, salt container lid, spray arms, etc.). The same tendency reported for pre-wash experiments is observed in intensive washing: the total MP concentration decreased from 96 MPs/L to a stable value of 76–75 MPs/L, while tap water contributed 5–10 MPs/L (Figure S4a).

Kelly et al. (2019) found that the highest microfibre release was obtained in a laundry when the highest volume of water was used, which is related to the programme length. This also agrees with previous results reported by Periyasamy (2021), who indicated that washing jeans at higher temperatures and with longer wash times implied a higher release of microfibres into the environment. In the present study, it was observed that the average concentration of MPs measured in the washing effluent in the experiments M24, M25, N2, N3 and N8 (after stabilization) was 69.90 ± 1.10 MPs/L and 64.77 ± 1.00 MPs/L for intensive and pre-wash programmes, respectively. In respect of MP concentrations, this difference confirms the slight effect of temperature because the intensive cycle uses a higher volume than the pre-wash programme. Nevertheless, when the total number of MPs released is considered, this effect cannot be overlooked. Certainly, tap water contributed 16–31 MPs and 62–144 MPs in pre-wash and intensive programmes, respectively. When this contribution is subtracted from total number of MPs emitted in the pre-wash (272 ± 85 MPs) and intensive cycles (1166 ± 143 MPs) to show the MPs that can be attributed to the dishwasher, the result implies a higher release of MPs in the intensive programme than in the pre-wash, which is explained by the temperature effect, showing that higher temperature leads to more thermal degradation of plastics (Lin et al. 2022). The plastic material of the tub may be degraded by the heat emitted by the electrical resistance heating element of the dishwasher (Venkatesh 2022). Obviously, a longer programme implies a greater volume of water and therefore a higher release of MPs. Moreover, a linear correlation between the number of MPs and the volume of water used could not be established (Figure S5).

In addition, MPs have been classified according to size, shape, colour and chemical composition (Figure S6). When MPs from dishwasher effluent were compared to those in the tap water samples, some of the differences are important. In terms of shape, it can be observed that the abundance of fragments increased both in pre-wash (77%) and in intensive wash (79%), compared to tap water (60%), whereas the abundance of fibres decreased from 36% in tap water to 14% in pre-wash and intensive. This is comprehensible, since the plastic inside the dishwasher is made of polypropylene granulate reinforced with 20% talcum and the washing process, especially when high temperature, (75 °C) was used, producing mechanical abrasion that favoured the release of fragments (Zhang et al. 2021). In tap water, 45% of MPs were smaller than 250 μm, whereas this proportion increased in pre-wash (63%) and intensive (67%). This is in agreement with observations of Song et al. (2017), who reported an increase in the number of fragmented polymer particles when particle size decreased. As mentioned in ‘Materials and methods’, several parts of the dishwasher are grey in colour, except the upper and lower spray arms, which are purple. In tap water, grey is the colour of 40% of the total MPs, whereas in pre-wash and intensive, this value increases to 72% and 76%, respectively. In addition, no purple colour was identified in tap water, but many purple MPs were observed in the effluent from washing programmes, representing 11% and 19% in intensive and pre-wash programmes, respectively. So, it is indicative of MP origin. This proves the emission of MPs by degradation of dishwasher plastics. Regarding the chemical composition, the percentage of PP increased from 36% in the tap water to 94–95% in the washing effluents. The above-mentioned data therefore highlight the fact that this conventional household appliance can act as a notable source of MPs.

### Release of MPs by plastic lunch boxes

It is frequent that some of the utensils washed in the dishwasher machine are made of plastic. In particular, lunch boxes used in food storage are usually made from glass or PP, although other polymers can be also used. To evaluate the release of MPs during washing of plastic lunch boxes, pre-wash and intensive wash experiments were carried out. These tests were carried out in March and November of 2021 (see Table 1).

In addition to the MPs released from the tap water and dishwasher, in this case, a third source of MPs was added. Thus, in order to quantify the MPs coming from lunch boxes, it is necessary to know the number of MPs coming from the tap water and those released from the dishwasher. As can be observed in Table 1, before each experiment with lunch boxes, the tap water was analysed and experiments with the empty machine were carried out. So, the difference between the total number of MPs in the washing effluent and the number of MPs coming from tap water corresponds to the sum of the MPs emitted by the dishwasher and plastic lunch boxes. Then, the number of MPs emitted by the dishwasher was considered to be the same as that obtained in the same experiment carried out with the empty dishwasher and the number of MPs emitted by the lunch boxes was calculated by difference.

As can be observed in Fig. 2b, the number of total MPs emitted from the dishwasher increased by 61–93 MPs (81 ± 18 MPs) and 875–1055 MPs (996 ± 64 MPs) in pre-wash and intensive programmes, respectively, in comparison to the respective washings carried out with the empty dishwasher. This increase corresponds to the contribution of the six plastic lunch boxes, with mean contributions of 14 ± 3 MPs and 166 ± 12 MPs per lunch box unit in pre-wash and intensive, respectively. Again, it was observed that more MPs were released from plastic lunch boxes with a longer washing time and higher temperature, factors which increase MP release by abrasion. This is reinforced by the fact that the numbers of MPs emitted in the pre-wash and intensive programmes were 105–89 MPs/L and 152–130 MPs/L, respectively (Figure S4b). These values were much higher than those recorded when the washing was done with the equipment running empty (Figure S4a), which was due to the MPs released from the lunch boxes, which emitted on average 26 ± 6 MPs/L (pre-wash) and 64 ± 9 MPs/L (intensive), with a contribution of 4 ± 1 MPs/L and 11 ± 2 MPs/L per plastic container unit in pre-wash and intensive programmes, respectively.

Du et al. (2020) analysed the emission of MPs from take-out food containers made of PP, PS, PE and PET. A treatment by immersion in hot water and shaking was evaluated, and it was reported that PP containers were the most resistant to abrasion; hence, only between 3 and 9 MPs (≥ 43 μm) were released per take-out food container. Moreover, these authors found no significant differences between this treatment and direct washing at room water temperature of the inner surface of the container, concluding that temperature did not play a major role in this case. The MP release observed in the present work was significantly higher, even for the mildest programme, due to the mechanical abrasion caused by the high-pressure water jetting used by the dishwasher. Other authors have reported that the use of hot water (100 °C) helps in the release of MPs in food and drink containers (Hee et al. 2022; Liu et al. 2022).

Figure S7 shows the main characteristics of MPs found in the effluents of pre-wash and intensive programmes when the six PP lunch boxes were washed. For pre-wash, MPs smaller than 250 μm represented 56% of total, a value slightly lower than that found for empty pre- (63%) and intensive (67%) washing. This abundance was higher when the intensive programme was used (70%). It seems that the use of more severe conditions promoted the fragmentation of the released polymer particles (Song et al. 2017). In all cases, fragments were the predominant shape, increasing from 77–79% with empty washings to 88–90% when lunch boxes were washed. In addition, the emission of more PP particles due to the presence of lunch boxes implies that the abundance of this polymer type in the final mixture increased till it amounted to 96% of the total. It is noteworthy that the frequency of blue colour increased from 1–2% for empty washing to 7–8% when lunch boxes were washed, mainly because the lids of the plastic lunch boxes are of this colour.

### Effect of detergent on MP release

In order to discover the contribution of the MPs contained in commercial detergents to the MP content of the dishwashing effluents, MP concentration, colour, shape and chemical composition were evaluated in three different commercial brands of dishwasher detergents.

As can be seen in Figure S8, 3.02 ± 0.33 MPs/g, 1.48 ± 0.39 MPs/g and 0.75 ± 0.34 MPs/g were found in detergents 1 (cube), 2 (cube) and 3 (pod), respectively. Bayo et al. (2022) analysed 19 commercial brands of dishwashing detergents, finding lower values than those obtained in this study. The weight of each cube/pod was between 16 and 18 g, so detergent contributes between 12 and 52 MPs in each washing programme. This is a contribution similar to that of the tap water in pre-washing, but much lower than the number of MPs released from dishwasher accessories and lunch boxes. The most predominant shapes found in the three detergent samples were fragments (57–72%), and PP was the most common chemical composition (63–87%). Predominant colours were different for the three detergent brands.

For analysing the possible effect of the detergent on both the release of MPs from the dishwasher and the plastic lunch boxes, detergent 1 was used. As is shown in Fig. 2a, on the dates N2, N3 and N8, between 1025 and 1030 MPs (1028 ± 3 MPs) were emitted using the intensive programme in the empty dishwasher and without detergent. When the same washing programme was repeated on the same dates, but using detergent, the total release of MPs was between 1382 and 1591 MPs (1503 ± 108 MPs) (Fig. 2c). This implies that detergent promotes the release of polypropylene MPs from dishwasher accessories, increasing the number of MPs released by 35–54%. This means that MP concentration in the effluent from the intensive programme without detergent was 70 ± 1 MPs/L (Figure S4a), whereas when detergent was used, this value increased to 100 ± 3 MPs/L (Figure S4c). According to Periyasamy (2021), due to their alkaline nature, detergents induce chemical damages in jeans. This effect increased when jeans were exposed to higher washing temperatures and longer washing times. In a similar way, in the present work, the polypropylene components of the dishwasher are susceptible to oxidative attack by the detergent, which could cause the release of a higher number of microplastics.

The potential discharge of MPs in a small city due to the use of dishwashers is shown in the following example. According to the Spanish National Statistics Institute (INE), a city of 217,552 inhabitants such as Oviedo (Asturias, Spain) has 221,507 dwellings (INE 2022). Considering the data shown above, if each household carries out one wash per day, using an intensive programme with similar characteristics, around 2.27 × 10^8^ MPs per day are emitted when the washing is done with the empty dishwasher. This value increases to 3.21 × 10^8^ MPs per day if a detergent tablet is added to the dishwasher. These microplastics are released into the sewage system, reaching a wastewater treatment plant (WWTP) that is capable of removing up to 90% of the MPs. However, this removal efficiency is insufficient, as a large number of MPs escape into the environment (Masiá et al. 2020; Sol et al. 2020, 2021).

In addition to the influence of the detergent on MP release from the plastic parts of the dishwasher, the possible effect of the detergent on plastic lunch box degradation has also been analysed and results are shown in Fig. 2d. As in previous experiments, the number of MPs released from lunch boxes was estimated by comparison with the experiments carried out with the empty dishwasher. So, when six PP lunch boxes were washed using the intensive programme, without detergent (dates N2, N3 and N8), 999–1042 MPs (1032 ± 29 MPs) were released from lunch boxes. Likewise, when detergent was used under the same conditions, the emission of MPs from lunch boxes was almost the same, ranging between 900 and 1039 MPs (966 ± 70 MPs) (Fig. 2d). Comparing these values in terms of MP concentrations, when these experiments are carried out, 181–169 MPs/L are released (Figure S4d), this figure being higher than in the case of intensive washing (114–95 MPs/L) (with detergent and without lunch boxes) (Figure S4c).

Although the process is not the same, other authors have found that the use of detergents can favour a greater release of microfibres during textile washing, the number of microfibres increasing by up to 300% (De Falco et al. 2018a, b; Hernandez et al. 2017; Napper and Thompson 2016; Periyasamy 2021; Yang et al. 2019). In this case, the presence of detergent increased the release of MPs from dishwasher accessories, but it did not affect the erosion suffered by the PP lunch boxes. This may be due to the specific type of plastic, which was not exactly the same, since the dishwasher is made of polypropylene reinforced with 20% talcum (PP 20 T), whereas the lunch boxes consist of polypropylene, which implies the lower resistance of the former to degradation by detergent.

The main characteristics of the MPs found in the experiments carried out with detergent with the empty dishwasher and containing lunch boxes are shown in Figures S9 and S10. In both cases, MPs < 250 μm were still the most common, accounting for 61–67% of the total. In addition, the vast majority of the microplastic particles were fragments, these representing 84–89% of the total. Additionally, the polypropylene particles were also the most abundant in both cases (95%), due to the degradation of the plastic inside the dishwasher. This value was very similar to that observed in the two previous cases, washings without detergent.

## Conclusions

In the present study, dishwashing has been experimentally evaluated as a source of MPs released into urban wastewater. Two programmes have been investigated, and it was found that the pre-wash cycle (15 min and room water temperature) released 207–427 MPs, whereas 1025–1370 MPs were released in the intensive cycle (164 min and 70 °C). Results showed that plastic materials inside the dishwasher can be degraded during the washing processes, which increases the number of MPs discharged into the sewage system, and therefore, dishwashing, like laundering, can be considered an important source of microplastic pollution at household level. In addition, when plastic food containers are washed, the number of MPs released increased by 14 ± 3 MPs and 166 ± 12 MPs per lunch box unit in pre-wash and intensive programmes, respectively. This indicates that using glass lunch boxes instead of plastic ones can help to reduce the MPs discharged to WWTPs. Moreover, as has been reported for washing processes of synthetic textiles in domestic washing machines, the higher the temperature and the longer the time of exposure, the greater is the release of MPs from the plastic dishwasher constituents and the lunch boxes. In addition, it has been found that the use of detergent increased the degradation of plastic dishwasher accessories by 35–54%. On the contrary, the detergent did not affect the release of MPs from lunch boxes due to the different compositions of the PP in the dishwasher and the food containers, which reflects the influence of the nature of each plastic on degradation.

To sum up, it is important to remark that this work contributes to enhancing our knowledge about MPs released during household activities, which relates to the interventions of the European Union designed to encourage and enforce the limitation of single-use plastic products as well as products containing microplastics. Furthermore, this work is relevant to a proposal under consideration about microplastic pollution in treated water and sewage sludge that seeks to reduce the dispersion of these microparticles (Sol et al. 2020).

## Supplementary Information

Below is the link to the electronic supplementary material.

Supplementary file1 (DOCX 7.23 MB)

## Data Availability

The datasets used and/or analysed during the current study are available from the corresponding author on reasonable request.
