# Supplementary material for: Contribution of household dishwashing to microplastic pollution
Source: Environ Sci Pollut Res Int. 2023 Jan 26;30(15):45140–50. doi: 10.1007/s11356-023-25433-7 (PMC10076389; doi:10.1007/s11356-023-25433-7)
Supplement: Supplementary file 1 — Supplementary file1 (DOCX 7.23 MB) [file 11356_2023_25433_MOESM1_ESM.docx]

**Supplementary Materials**

Contribution of household dishwashing to microplastic pollution

Daniel Sol, Andrea Menéndez-Manjón, Sofía Carrasco, Jacinto Crisóstomo-Miranda, Amanda Laca, Adriana Laca*, Mario Díaz.

*Department of Chemical and Environmental Engineering, University of Oviedo, C/Julián Clavería s/n, 33006, Oviedo, Spain.*

* Corresponding author ([lacaadriana@uniovi.es](mailto:lacaadriana@uniovi.es))


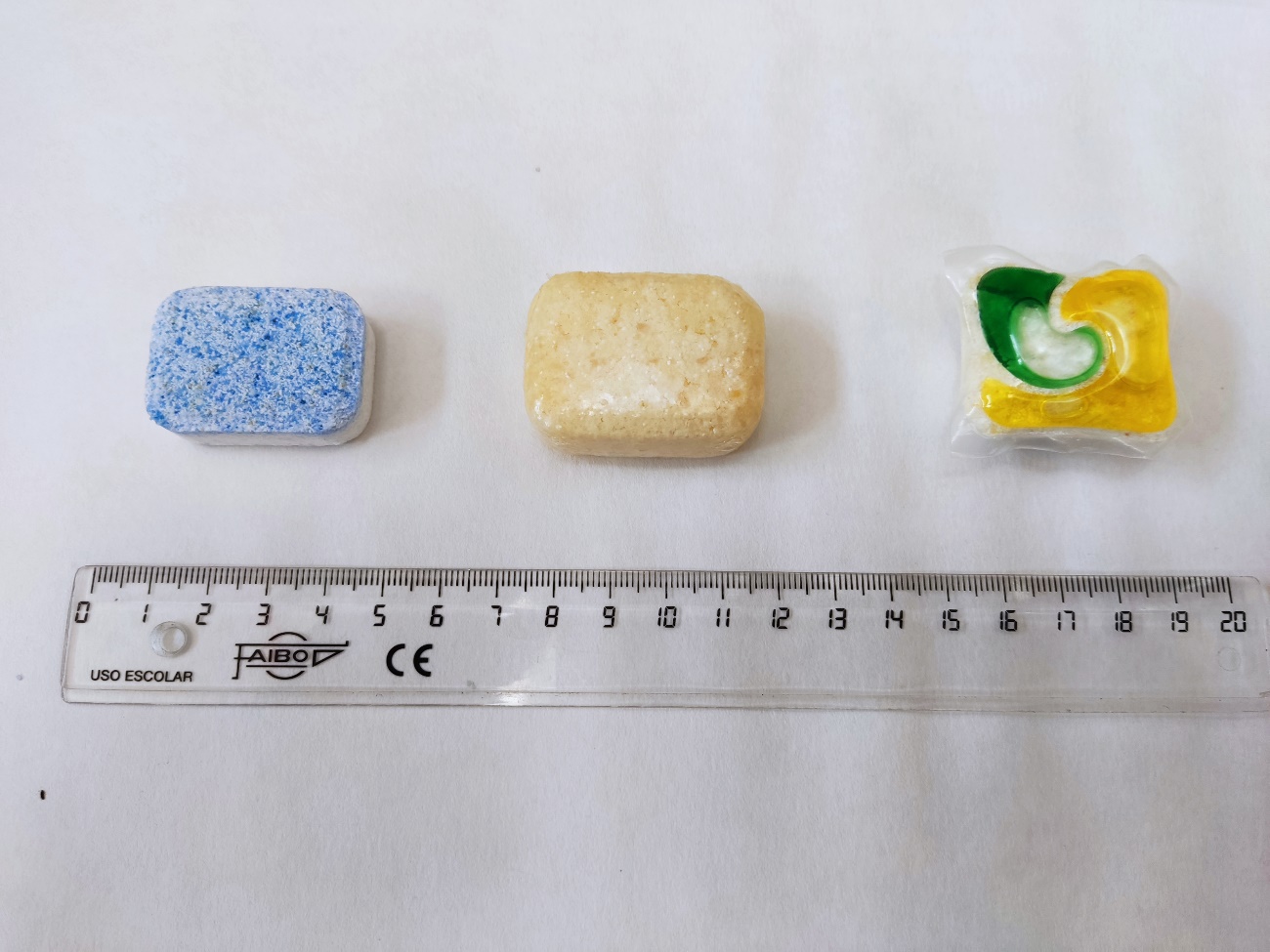


**Figure S1.** Dishwashing detergents analysed in the present study. From left to right: Finish (detergent 1), Presto (detergent 2) and Fairy (detergent 3).


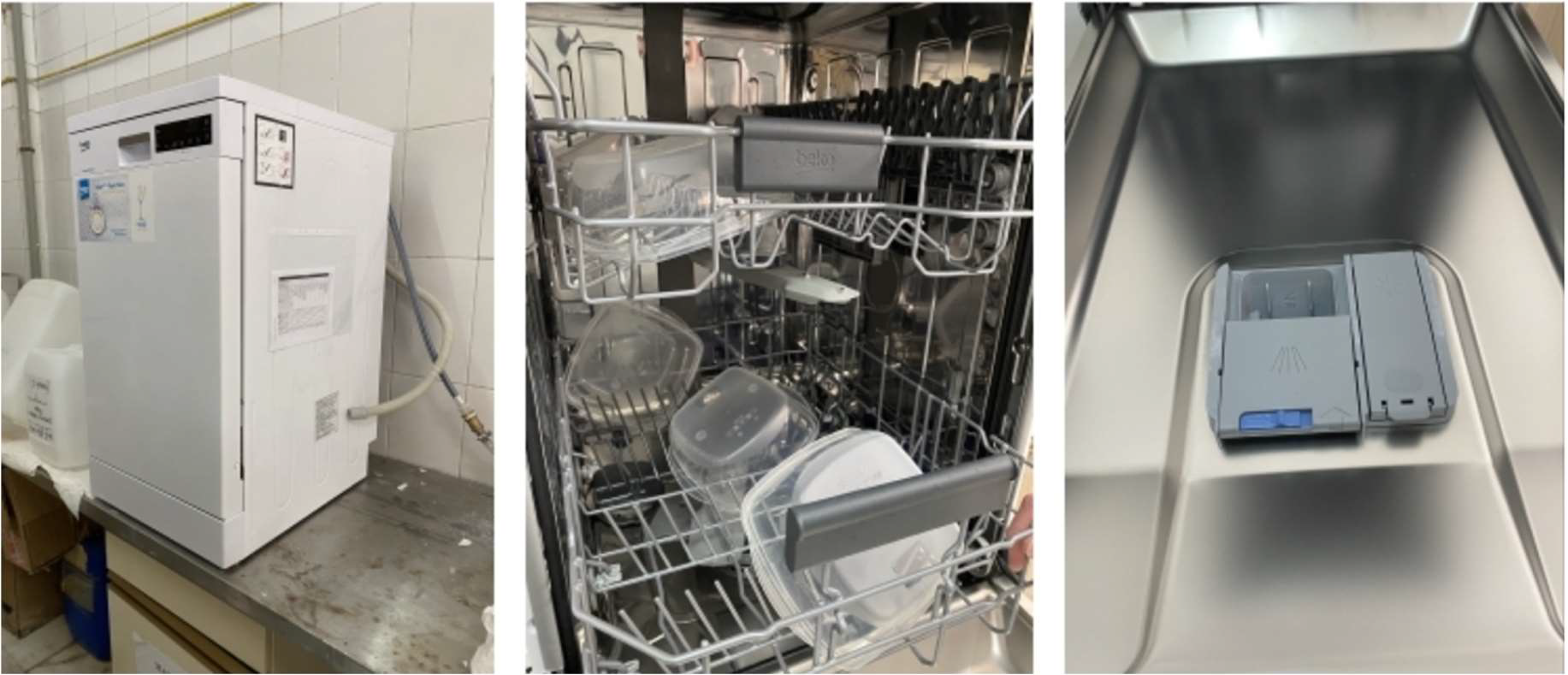


**Figure S2.** Medium load dishwasher (Beko DFS28021W) made of stainless steel and polypropylene reinforced with 20% Talcum (PP 20T) employed in this work.


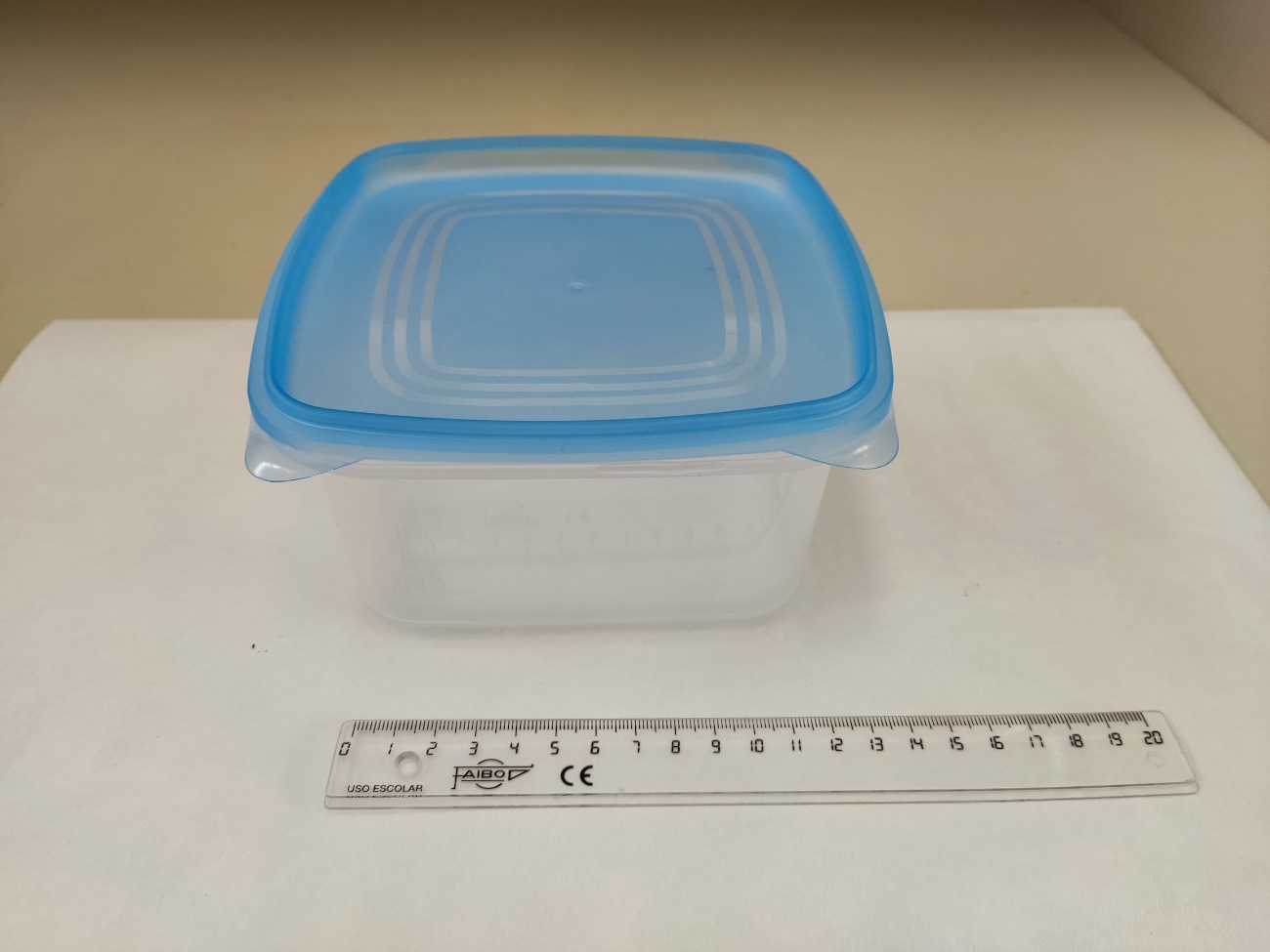


**Figure S3.** Square plastic lunch boxes, with a capacity of 0.75 L and dimensions of 12x12x8 cm, made of polypropylene, used in the present study.

**
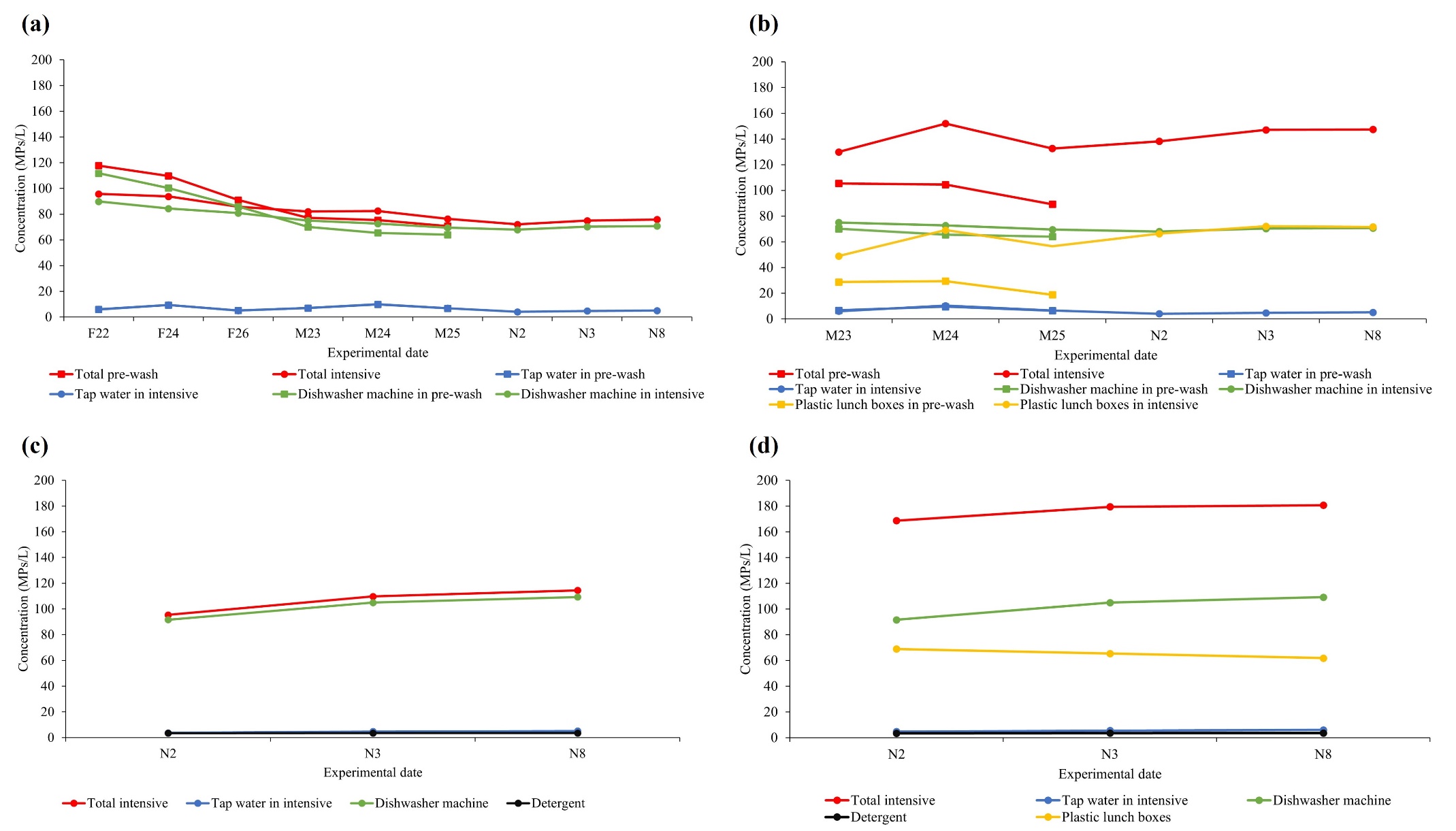
Figure S4.** MP concentrations emitted during the different experiments carried out, (a) empty dishwasher machine, (b) with plastic lunch boxes, (c) empty dishwasher machine with detergent and (d) with detergent and plastic lunch boxes. Samples were taken in 2021 (F22, F24 and F26 in February; M23, M24 and M25 in March; N2, N3 and N8 in November).

**Figure S5.** Number of MPs emitted by volume of washing, (a) pre-washing; (b) intensive programme.

**Figure S6.** Main characteristics of MPs found in pre-wash and intensive programmes according to the size, shape, chemical composition and colour (average values are shown).

**Figure S7.** Main characteristics according to the size, shape, chemical composition and colour of MPs found when six plastic lunch boxes were washed in pre-wash and intensive programmes (average values are shown).

**Figure S8.** Main characteristics of MPs found in detergents according to the MP concentration (a); shape (b); colour (c) and (d) chemical composition. The inner, intermediate, and outer ring refer to detergents 1, 2 and 3, respectively.

**Figure S9.** Main characteristics of MPs found according to the size, shape, chemical composition and colour in intensive program when detergent was used (average values are shown).

**Figure S10.** Main characteristics of MPs found according to the size, shape, chemical composition and colour when detergent is used to wash plastic lunch boxes in intensive program (average values are shown).
